# Hypodermic Treatment of Diseases

**Published:** 1867-01

**Authors:** 


*Hypodermic Treatment of Diseases.*—Prof. Gibbons, of San Francisco, remarks: "In the choice of a syringe, the simplest construction is the best. I have found that those made of gutta percha, with the canula fitting on without screwing, are least liable to get out of order or broken. It is not safe to depend on the graduation marked on the syringe; in some of the instruments the divisions do not correspond to halves and quarters of drachms. Every operator should carefully test his own syringe in this respect.

"The pain is trifling in general, if the operation be dexterously performed. The skin should be sharply pinched, as well to cover the prick of the instrument as to secure the hold. To have the fold of skin slip from the fingers is awkward. If the surface be greasy or sweaty a fold of rag should be interposed. These are small matters, but they are worthy of attention. The liquid should not be too rapidly injected, else the cells of the subcutaneous tissue are torn. It is well to avoid the veins. The instrument may be withdrawn by a sudden jerk, and the finger pressed on the spot for a moment. This is scarcely necessary, however, as I have never seen the liquid escape from the orifice after being injected. There is no need of rubbing the part with the finger, as some propose, to diffuse the injected liquid and promote its absorption; and the idea of always injecting it downward to prevent it from gravitating out of the puncture is sheer nonsense.

"In some parts of the body the puncture is less painful than in others. The dorsal surfaces of the arm and forearm are not very sensitive, and present very convenient sites for the application. I am partial to the region about the insertion of the deltoid muscle. The outside of the thigh, the calf of the leg, and the region of the spine admit of the operation without much pain. The skin of the chest and abdomen is very tough, and patients also complain much of pain from the puncture in this region. The operation is painful, and sometimes followed by inflammation, on the hands and feet, and these parts should be avoided. I have seen no evil results from puncture of the scalp or behind the ears in neuralgia affecting those regions. Where, however, the skin is bound down closely to the bone beneath, the quantity of fluid injected should not be large, and it should be introduced very slowly to avoid painful distension. In emaciated subjects the skin becomes very thin and as tough as leather, rendering it difficult of penetration. This fact should be kept in view, in order to avoid failure in the first attempt; for it is important always to make sure of introducing the instruments through the skin at once, by a sudden plunge. Otherwise, unnecessary pain is produced, and the patient may refuse again to submit to the operation. Large-sized cannula gives much more pain than a small one. In general, when complaint is made the puncture is alone complained of. But sometimes the distention produced by the liquid is painful, especially if it be suddenly injected, or if the quantity be large. In very sensitives subjects it is well to warm the liquid."

